# Salter-Harris II injury of the proximal tibial epiphysis with both vascular compromise and compartment syndrome: a case report

**DOI:** 10.1186/1749-799X-4-23

**Published:** 2009-06-29

**Authors:** Nicholas D Clement, Anukul Goswami

**Affiliations:** 1 Dept of Trauma and Orthopaedic Surgery, Royal Infirmary of Edinburgh, Little France, Edinburgh, EH16 4SU, UK; 2 Borders General Hospital, Melrose, TD6 9BS, UK; 3 17 Weybourne Lea, Eastshore Village, Seaham, SR7 7WE, UK

## Abstract

We present a case of a Salter-Harris II injury to the proximal tibia associated with both vascular compromise and compartment syndrome. The potential complications of this injury are limb threatening and the neurovasular status of the limb should be continually monitored. Maintaining anatomic reduction is difficult and fixation may be needed to achieve optimal results.

## Introduction

Salter-Harris injuries of the proximal tibia are rare, with an incidence of 0.5 to 3% of all epiphyseal injuries[1,2]. This rarity is due to the anatomy of the proximal epiphysis; the collateral ligaments insert distally into the metaphysis shielding the epiphysis. There have been limited reports of these injuries to date, with the largest published series reporting 39 cases [3]. This injury is potentially limb threatening, secondary to vascular compromise or compartment syndrome [4].

We report a posteriorly displaced Salter-Harris II injury to the proximal tibia associated with both vascular compromise and compartment syndrome.

## Case report

A 14-year-old girl presented to our accident and emergency department after sustaining a direct blow from a fence post to the anterior aspect of her proximal tibia whilst riding her horse at approximately 15 km/hr. She then fell to the ground, forcing the knee into valgus. She was unable to weight bear because of pain localised to the knee.

On examination her right knee was deformed, with a step inferior to the joint margin. The leg was also externally rotated by 20 degrees. There was marked tenderness over the proximal tibia. The calf was soft and non-tender; peripheral pulses and neurology were intact.

**Figure 1:**
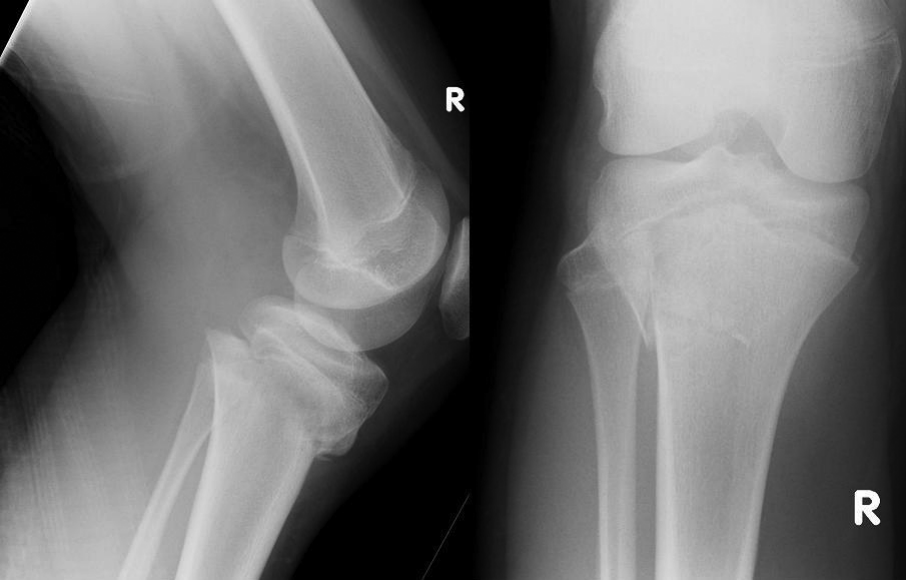
**Pre-operative radiographs**.

**Figure 2:**
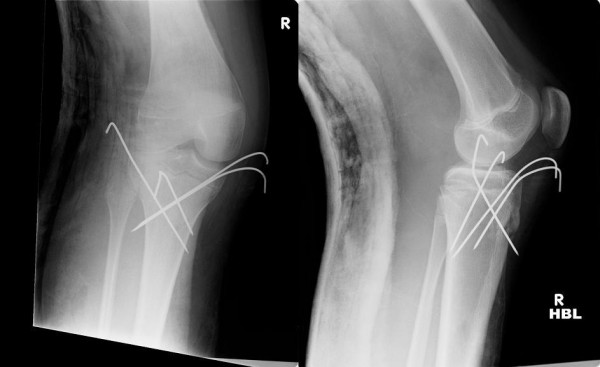
**Immediate post-operative radiographs**.

Radiographs revealed a Salter-Harris II injury, with a lateral metaphyseal extension and posterior displacement of the tibia (Figure 1). She was then taken to theatre within 5 hours of presentation, however at this time she complained of "pins and needles" over the dorsum of her foot. The pulses were re-examined, and found to be absent. Under general anaesthetic the fracture was reduced. This was achieved with forward traction over the proximal tibia distal to the epiphysis, with the knee flexed to 100 degrees. On reduction the peripheral pulses returned but remained weak. The fracture remained unstable and continued to fall back to its original position with loss of pulses on release of traction. Reduction was held with four Kirschner (K-) wires (Figure 2).

Despite fixation the pulse remained barely palpable. The calf was tense. Anterior compartment pressure measured at 55 mmHg. All four compartments were decompressed with fasciotomies. Vascularity of the limb was immediately restored and confirmed with a portable Doppler instrument. An above knee back slab was applied in 45 degrees of flexion at the knee. The fasciotmies were closed over next seven days in three stages.

**Figure 3:**
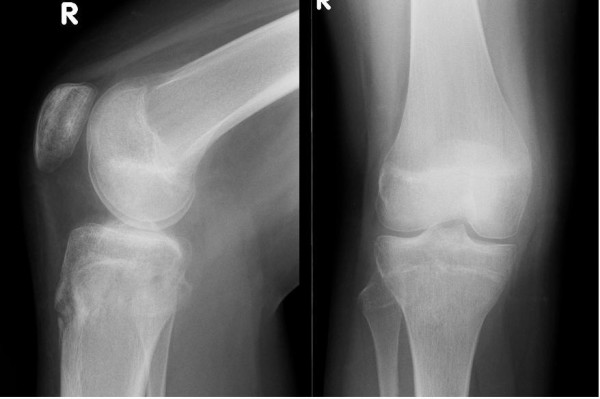
**Six months post-operative radiographs**.

The cast and wires were removed at 6 weeks, during which time she was not allowed to weight bear on the affected limb. Between 6 to 12 weeks she was allowed partial to full weight bearing under physiotherapy supervision. At last review, 1 year post injury; there was no deformity, instability or leg length discrepancy. Radiographs at this point demonstrated healing of the fracture (Figure 3).

## Discussion

This is the first reported case with both vascular compromise and compartment syndrome secondary to a proximal tibial Salter-Harris injury.

An epidemiological study of epiphyseal growth plate injuries demonstrated an incidence of 0.5% [1]. Burkhart et al reported a higher incidence of 3.06% from the Mayo Clinic, which may represent the referral pattern to this specialist centre [2]. The majority cases are male, and are Type II injuries with a peak incidence is between 12 and 14 yrs (Table 1) [2-10].

**Table 1:** Epidemiology and mechanism of Salter-Harris injuries to the proximal tibia.

**Author *et al***	**Fracture Number**	**Patient Number**	**% Male**	**Mean Age (yrs)**	**Cause of Injury**			
					
					**Sports**	**RTA**	**Bicycle**	**Other**
Aitkin (1956) [5]	2	2	100	11	1	1	0	0

Shelton (1979) [3]	39	38	97	14	18	12	4	4

Burkhart (1979) [2]	28	27	85	11	11	8	1	7

Bertin (1983) [6]	13	13	Unknown	14	2	11	0	0

Gill (1984) [9]	3	3	100	15	0	2	0	1

Poulsen (1989) [7]	15	15	73	15	2	13	0	0

Wozasek (1991) [4]	29	29	67	13	12	11	0	6

Gautier (1998) [10]	6	6	83	11	1	1	0	4

Rhemrev (2000) [8]	6	6	67	13	1	1	0	4

**Totals**	141	139	84	13	48 35%	60 43%	5 4%	26 19%

The described mechanism of injury is direct impact to the proximal tibia with the knee in extension or hyperextension, with or without valgus or varus strain [5]. The cause of injury varies (Table 1). A recent case report, however describes minor trauma in an obese adolescent sustaining consecutive bilateral proximal tibial fractures, which may suggest an associated change at physeal closure predisposing to Salter-harris injuries [11]. Bertin et al demonstrated associated ligament injuries with these injuries, reporting 13 cases of which 8 (62%) had associated ligamentous injures (anterior cruciate (ACL) 4, medial collateral 3 and both 1) [6]. Poulsen et al also illustrated similar ligamentous injuries, with 5 out of 15 patient suffering ACL injuries [7].

The first reported case of vascular compromise was published in 1894 [12]. Ten cases since have been published as part of a case series (Table 2) [2-4,6,9,10]. Five of these ten patients had posterior displacement, of which three went onto develop gangrene. This was due to a delayed diagnosis; with a normal peripheral pulse being on admission, but then subsequently lost and not reassessed [2]. Only two cases of compartment syndrome have been reported (Table 2) [2,3]. Our case was also posteriorly displaced, and demonstrated delayed vascular compromise. The associated compartment syndrome, we believe was secondary to the injury and not due to the vascular deficit, because the period of compromise was minimal, and it would have occurred later after reperfusion.

**Table 2:** Salter-Harris classification and complications of injuries to the proximal tibia.

**Author *et al***	**N^o ^Salter-Harris**						**VC**	**AM**	**CS**
				
	0*	**I**	**II**	**III**	**IV**	**V**			
Aitkin (1956) [5]	0	0	1	1	0	0	0	0	0

Shelton (1979) [3]	0	9	17	10	3	0	2	2	1

Burkhart (1979) [2]	0	3	9	6	8	2	1	1	1

Bertin (1983) [6]	0	1	7	4	1	0	1	0	0

Gill (1984) [9]	0	0	2	1	0	0	1	1	0

Poulsen (1989) [7]	0	0	4	4	6	1	0	0	0

Wozasek (1991) [4]	8	5	11	4	1	0	4	1	0

Gautier (1998) [10]	0	3	0	1	2	0	1	0	0

Rhemrev (2000) [8]	0	1	1	2	2	0	0	0	0

**Totals**	8 6%	22 16%	52 37%	33 23%	23 16%	3 2%	10 7%	4 3%	2 1%

*Wozasek et al classified tenderness at the epiphysis and impaired knee joint function with normal radiograph findings as type 0. VC = Vascular Compromise AM = Amputation CS = Compartment syndrome

A common theme throughout the literature is the difficulty in maintaining the reduction with cast alone, especially with posterior displacement of the tibia [2-10]. The majority of reports used conservative measures for displaced type I and II (MUA and cast in varying degrees of flexion) and open reduction and internal fixation of displaced type III, IV and V. Some authors regret not fixing type I and II fractures, with subsequent loss of reduction and unsatisfactory outcomes [8]. The reported case needed supplementary K-wires to maintain reduction due to the instability and vascular compromise.

Proximal tibial epiphyseal injuries differ from the Salter and Harris' generalised prognosis [13]. Shelton defined an unsatisfactory outcome as: leg length discrepancy of 25 mm or more and/or angular deformity of more than 7 degrees.3 A high percentage of type I and II injuries result in an unsatisfactory outcome (Table 3), which is probably related to growth disturbance of the physis after epiphyseal separation [14]. In contrast growth disturbance is limited in Salter-Harris III and IV injuries as epiphyseal separation does not occur [15], with minimal insult to the physis resulting in better outcomes relative to type I and II injuries. Although, in part this may also reflect the difficulty in maintaining the reduction with cast alone, as this was used in the majority of type I and II injuries and could have contributed to the poor outcomes in this group.

**Table 3:** Outcomes after injury.

**Author *et al (yr published)***	**Salter-Harris Type**	**Number**	**Satisfactory**	**Unsatisfactory **(>24 mm/>5^o^)
Aitkin (1956) [5]	II	1	1	-
	III	1	1	-

Shelton (1979) [3]	I	9	6	3
	II	17	12	5
	III	10	9	1
	IV	3	3	-

Burkhart (1979) [2]	I	3	2	1
	II	9	8	1
	III	6	6	-
	IV	8	3	5
	V	2	2	-

Bertin (1983) [6]	I	1	-	1
	II	7	6	1
	III	4	1	3
	IV	1	1	-

Gill (1984)9	No long-term follow up			

Poulsen (1989) [7]	II	4	4	-
	III	4	4	-
	IV	6	4	2
	V	1	1	-

Wozasek (1991) [4]	No Type specific breakdown, but out of the 23 patients reviewed 17 (74%) had a satisfactory outcome			

Gautier (1998) [10]	I	3	2	1
	II	0	-	-
	III	1	1	-
	IV	2	1	1

Rhemrev (2000) [8]	I	1	-	1
	II	1	1	-
	III	2	2	-
	IV	2	2	-

**Subtotals**	I	17	10 (59%)	7 (41%)
	II	39	32 (82%)	7 (18%)
	III	28	24 (86%)	4 (14%)
	IV	22	14 (64%)	8 (36%)
	V	3	3 (100%)	0

**Totals**	I–V	109	83 (76%)	26 (24%)

## Conclusion

Fractures of the proximal tibial epiphysis are rare, and the potential complications in this young population are limb threatening. Constant monitoring of neurovascular status is essential to identify acute and delayed compromise. A low tolerance should be taken to use supplementary fixation, such as K-wires, in view of the difficulty in maintaining the reduction and the potential for poor outcomes should this be lost.

## Competing interests

The authors declare that they have no competing interests.

## Authors' contributions

AG was the surgeon in charge of the patient described with in this report. NC conducted the literature review and analysed the gathered reports for the described injury. NC composed and wrote the manuscript. Both authors read and approved the final manuscript.
